# Maternal cannabis use in pregnancy, perinatal outcomes, and cognitive development in offspring: a longitudinal analysis of the ALSPAC cohort using paternal cannabis use as a negative control exposure

**DOI:** 10.1007/s10654-025-01233-w

**Published:** 2025-05-12

**Authors:** Daniel J. Corsi, Tim T. Morris, Zoe E. Reed, George Davey Smith

**Affiliations:** 1 Faculty of Medicine, School of Epidemiology and Public Health, University of Ottawa, Ottawa, Canada; 2 CHEO Research Institute, CPCR Building, Rm L1132, 401 Smyth Road, Ottawa, ON K1H 8L1 Canada; 3 Centre for Longitudinal Studies, Social Research Institute, University College London, London, UK; 4 MRC Integrative Epidemiology Unit, University of Bristol, Bristol, UK; 5 School of Psychological Science, University of Bristol, Bristol, UK

**Keywords:** Pregnancy, Cannabis, Substance use, Perinatal, Cognitive development, Neurodevelopment, Epidemiology, ALSPAC

## Abstract

Maternal cannabis use in pregnancy is reported to be associated with perinatal and neurodevelopmental outcomes in offspring. Such associations, however, may be biased by residual confounding by socioeconomic position (SEP). To assess confounding, we use paternal cannabis use in pregnancy as a negative control exposure. We use data from 15,013 mother–father–child trios from the ALSPAC birth cohort, with participants initially recruited between 1990 and 1992. Exposures were maternal and paternal cannabis use during pregnancy. Neonatal anthropometry, perinatal, cognitive, and neurodevelopmental outcomes were modelled as a function of maternal and paternal cannabis use in pregnancy, adjusting for household-level SEP markers and maternal and paternal tobacco, alcohol, and drug use in pregnancy. We compared the strength of the association between maternal and paternal cannabis on outcomes using Wald tests. 5 and 13% of mothers and fathers reported cannabis use, which was inversely related to measures of SEP. Maternal cannabis use during pregnancy was associated with decreased infant birth weight (b = − 110.2 g, 95% CI − 185.1 to − 35.3 for any cannabis use) and length (b = − 0.45 cm, 95% CI − 0.84 to − 0.07). Maternal cannabis during pregnancy was also associated with neonatal special care admission (odds ratio 1.64, 95% CI 1.05 to 2.56) and lower education achievement scores at age 16 (b = − 19.2, 95% CI − 32.0 to − 6.3). Maternal cannabis use in pregnancy was modestly associated with perinatal outcomes and markers of cognitive development. However, most associations were attenuated after controlling for potential confounders, including SEP, and associations were not quantitatively different from paternal cannabis use. The association of maternal cannabis use in pregnancy with perinatal or cognitive outcomes in offspring may be driven by residual confounding, including SEP, rather than causal biological effects.

**Supplementary Information:**

The online version contains supplementary material available at 10.1007/s10654-025-01233-w.

## Introduction

Cannabis is the most used substance in pregnancy, after tobacco and alcohol [1]. The US National Survey on Drug Use and Health (2015–2019) found that 5% of pregnant respondents (n = 3657) reported any cannabis use [2], compared to 3% in the UK-based Avon Longitudinal Study of Parents and Children [3]. Cannabis use prevalence decreases throughout pregnancy, from 9.4% in the first trimester to 3.4% after the first trimester in the US survey, and from 5.2% preconception in Avon Longitudinal Study of Parents and Children (ALSPAC) to 2.2% in later pregnancy. Cannabis use in pregnancy varies substantially by demographic and socioeconomic factors, with younger women and those from socially disadvantaged families reporting higher use rates [4]. For example, an analysis of the birth registry in Ontario, Canada, found a prevalence of cannabis use of 10.6% among women aged 15–19 years in 2017–2018, compared to 0.6% among women aged 35 years and older, with rates increasing across all ages in recent years [4, 5].

Cannabinoids, including Δ^9^-tetrahydrocannabinol (THC), the psychoactive component of cannabis, directly impact the endocannabinoid system, a broad-spectrum modulator of the central and peripheral nervous systems [6]. Cannabinoid receptors are widespread throughout the body, including in various female reproductive tract tissues [7]. THC-induced dysfunction of the endocannabinoid system in pregnancy may impact placental function and increase the risk of adverse outcomes [8]. In addition, cannabinoids can cross the placenta, enter the fetal bloodstream, and may interact with the fetal endocannabinoid system, affecting neurodevelopment [9]. Importantly, the concentrations of Δ^9^-THC in cannabis plant material have risen substantially in recent decades [10], making it easier to consume higher doses of THC and potentially increasing adverse effects.

We previously demonstrated epidemiologic associations between maternal cannabis use and adverse pregnancy outcomes, including preterm birth and the need for neonatal intensive care, by analyzing birth registry data from Ontario, Canada [11]. Using a similar dataset and with follow-up of offspring for a median of 7 years, we found an association between maternal cannabis use during pregnancy and increased risk of offspring with autism and other neurodevelopmental conditions [12]. Offspring whose mothers had used cannabis during pregnancy had a higher prevalence of autism (2.2% compared to 1.4% in mothers who did not use cannabis). The associations were robust to adjustment for various potential confounders, including socioeconomic position (SEP) and substance use. Despite a comprehensive adjustment strategy, the inherent constraints of observational study designs frequently make it unlikely that residual confounding is eliminated.

Epidemiologic triangulation can help to draw more robust conclusions when using observational data [13, 14]. Using distinct analytical designs and datasets with different sources of confounding and bias may yield more reliable findings. This research is part of a broader effort to identify the role of prenatal cannabis use and its association with neurodevelopmental and cognitive outcomes using different designs [15, 16]. Here, we use ALSPAC, a UK birth cohort with a rich array of family-level lifestyle exposures and SEP indicators, to assess the strength of the association between maternal cannabis use and perinatal and cognitive outcomes. Specifically, we use paternal cannabis use during pregnancy, which is not available in the Ontario registry as a negative control exposure for maternal cannabis use [17, 18]. A negative control exposure approach assumes that if maternal cannabis use affects offspring outcomes via an intrauterine mechanism, then paternal cannabis use should not be independently related or at least substantially less robustly associated with those outcomes. Paternal cannabis use shares the same familial genetic and environmental confounding structure as maternal cannabis use, making it a suitable negative control exposure [19]. The outcomes of interest are newborn anthropometry, perinatal outcomes, hyperactivity at age 7, and cognitive and academic performance at ages 8 and 16. Although not strictly neurodevelopmental markers, these outcomes in ALSPAC provide sufficient variation for the present analysis and overlap with some dimensions of neurodevelopment. We hypothesize that because of shared household environment and confounding structures between mother and father [20], discordant maternal and paternal associations may indicate direct maternal biological effects. In contrast, concordant associations will be indicative of confounding effects. In addition, to examine the role of SEP as a confounding variable, we examine the socioeconomic patterning of cannabis use and outcomes in ALSPAC.

## Methods

Study participants were mother-partner-child trios from ALSPAC. Pregnant women who resided in Avon, UK, with expected delivery dates between April 1, 1991, and December 31, 1992, were invited to participate in ALSPAC [21, 22]. Partners, usually the biological father or father figure, were invited to participate in ALSPAC via the recruited mothers [23]. In the first recruitment phase, ALSPAC enrolled 14,676 fetuses antenatally or shortly after birth from 14,541 pregnancies during 1990–1992 [24]. Further enrolment of eligible families who wanted to participate but did not initially enroll began in 1999 when children were about 7 years of age for the Focus@7 assessment. Since this time, ALSPAC has conducted additional opportunistic and systematic recruitment of families, including offspring of index children. This has resulted in a main sample of 15,645 participants from 15,443 pregnancies. Details of the ALSPAC cohort and available data can be found in previous publications and on the study website through a searchable data dictionary and variable search tool (http://www.bristol.ac.uk/alspac/researchers/out-data/) [21, 22, 24]. Ethics approval was obtained from the ALSPAC Ethics and Law Committee and the Local Research Ethics Committees (http://www.bristol.ac.uk/alspac/researchers/research-ethics/). Informed consent for the use of data collected via questionnaires and clinics was obtained from participants following the recommendations of the ALSPAC Ethics and Law Committee at the time. At age 18, study children were sent 'fair processing' materials describing ALSPAC’s intended use of their health and administrative records and were given clear means to consent or object via a written form. Data were not extracted for participants who objected, or who were not sent fair processing materials.

### Exposure

Cannabis use by mothers and their partners was collected through self-reports in questionnaires posted to mothers and partners at 18–20 weeks’ gestation. For mothers, the questions were, “How often did you smoke marijuana/grass/cannabis/ganja—(a) in the 6 months before you conceived, (b) in the first 3 months of pregnancy, and (c) between 3 months and now.” Response options for each sub-question were “Every day, 2–4 times a week, once a week, less than once a week, not at all.” For partners, the questions were, “Please indicate how often you smoked marijuana/grass/cannabis/ganja—(a) in the 6 months before your partner conceived, and (b) in the last 3 months”, with response options for each being “Almost every day, two to four times a week, once a week, less than once a week, not at all.”

We have two markers as the primary exposure describing the frequency of use and where comparable questions were available for mothers and fathers. First, we categorized maternal and paternal cannabis, combining preconception and pregnancy use with a focus on the frequency of use: infrequent (less than 1 time a week), weekly (1 time per week), and more than weekly use (daily or 2–4 times a week) vs. no use. In addition, secondary binary exposure variables were created for mothers and partners, coded as any reported cannabis use, restricted to use during pregnancy vs. no use.

### Outcomes

We investigated perinatal, anthropometric, and cognitive and academic outcomes potentially associated with cannabis use in pregnancies. Perinatal outcomes include, for live births surviving the perinatal period, birthweight (g), birth length (cm), head circumference (cm), preterm delivery (less than 37 weeks’ gestation), and admission to a special care nursery. We also consider neonatal death. Anthropometric outcomes were selected for comparison with previous reports from ALSPAC [3]. For cognitive and academic assessments, we used performance in end-of-compulsory schooling education tests [General Certificate of Secondary Education (GCSE) capped points scores] from linked administrative data and total intelligence quotient (IQ) scores from the Wechsler Intelligence Scale for Children, administered at the 8-year clinic visits. GCSEs are education qualifications in a particular subject, typically around ages 15–16 in England, Wales, and Northern Ireland. As a marker of neurodevelopment related to hyperactivity and ADHD traits, we use the hyperactivity scale from the Strengths and Difficulties Questionnaire (SDQ), consisting of five items with scores varying between 0 and 10 and completed by the child’s teacher in school year 3 (age 7) [25].

### Covariates

Our covariate selection included robust measures of SEP, substance use, maternal BMI and psychopathology. We aimed to include a set of covariates to reduce confounding while focusing on the maternal–paternal cannabis use negative control comparison. We follow the guidance of Rothman and Greenland [26], the approach of previous studies using ALSPAC [27] and our prior work on cannabis use. We controlled for infant sex, parity, maternal age, maternal body mass index, maternal and paternal tobacco smoking in pregnancy, maternal and paternal alcohol use in pregnancy, maternal and paternal drug use in pregnancy, the highest level of parental education, the highest parental occupational social class, and the Cambridge Social Interaction and Stratification (CAMSIS) scale score (http://www.camsis.stir.ac.uk/) [28]. Maternal history of psychopathology is a composite measure comprised of maternal history of psychiatric problems, including severe depression, drug addiction, or alcoholism.

### Multiple imputation

Due to missing data in the variables of interest, listwise deletion reduced the complete case study population from 15,645 to 3905 (Fig. S1). Missing data patterns varied from 0% for sex to 49% for IQ measured at age 8. There was evidence that the missing data were patterned by exposures, outcomes, and covariates, suggesting the presence of non-random missingness that may bias complete case analyses (Table 1). To reduce the impact of this on our results, we used multiple imputation by chained equations in Stata (version 17) with 50 multiply imputed datasets. Before imputing, we excluded all individuals with missing data on sex at birth (n = 603) or month of delivery (n = 161). In cases where these data elements are missing, there is likely a major reason underlying the data recording, and these participants were excluded to reduce bias in the imputation. We examined the patterns of missingness on the imputation sample of 15,013 (Table S2). We included all remaining variables from the analytical models in the imputation models. We supplemented with additional variables for the delivery month, academic year, special educational needs, free meals eligibility, and IQ at age 15 to improve the accuracy and robustness of the imputed values (Table S3).

**Table 1:** Descriptive statistics from the complete case and multiple imputation samples, ALSPAC

	Complete case (n = 3905)							Imputed sample (n = 15,013)					
	No maternal cannabis			Any maternal cannabis			Missingness	No maternal cannabis			Any maternal cannabis		
	n	Mean or %	SD	n	Mean or %	SD	n (%)	n	Mean or %	SD	n	Mean or %	SD
Maternal age (years)	3748	28.9	4.5	157	26.6	5.3	1581 (10)	14,216	28.1	4.9	797	25.8	5.3
Partner age (years)	3748	30.7	5.3	157	28.8	5.9	5993 (38)	14,216	30.2	5.9	797	28.2	6.1
Primiparous	1862	49.7		94	59.9		2717 (17)	5798	40.8		415	52.1	
Maternal A level or higher	1534	41.0		76	48.4		3163 (20)	4752	33.4		313	39.3	
Partner A level or higher	1902	51.1		78	50.0		5719 (37)	5955	41.9		298	37.3	
Social Class I and II	968	25.8		47	29.9		4570 (29)	2572	18.1		125	15.7	
CAMSIS score (mother)	3333	56.6	12.8	139	56.4	14.5	6007 (38)	14,216	53.4	13.4	797	51.9	14.0
CAMSIS score (partner proxy)	3587	53.6	14.7	144	50.4	13.7	5268 (34)	14,216	50.4	14.6	797	46.9	14.0
Any paternal cannabis use	141	3.8		125	79.6		5833 (37)	1430	10.1		562	70.5	
Maternal cigarette smoking before pregnancy	929	24.8		129	82.2		2409 (15)	4528	31.9		635	79.6	
Maternal cigarette smoking in 1st trimester	662	17.7		96	61.5		2354 (15)	3992	28.1		543	68.1	
Partner cigarette smoking at start of pregnancy	1196	31.9		120	76.4		6115 (39)	4974	35.0		508	63.7	
Maternal alcohol consumption 1st trimester	555	14.8		47	29.9		2461 (16)	2533	17.8		247	30.9	
Partner alcohol consumption in pregnancy	2679	72.1		119	76.3		5806 (37)	10,628	74.8		618	77.5	
Maternal hard drugs in pregnancy	6	0.2		6	3.8		2558 (16)	50	0.3		54	6.8	
Partner hard drugs in pregnancy	41	1.1		23	14.6		5971 (38)	2950	20.8		307	38.5	
Maternal BMI (kg/m^2^)	3748	23.1	3.9	157	21.7	2.9	3989 (25)	14,216	23.0	3.9	797	21.6	3.3
Maternal history of psychopathology	321	8.6		37	23.6		3052 (20)	1472	10.4		175	21.9	

### Statistical analysis

We summarised the demographic, socioeconomic, and clinical characteristics in the complete case and imputed samples. We then examined cross-tabulations of maternal and paternal cannabis use in pregnancy to describe the most common patterns of parental cannabis use. All descriptive analyses were calculated across imputed datasets using Rubin’s rules, implemented by the mi prefix in Stata [29]. Our primary exposures of interest were the frequency of maternal and paternal cannabis use during pregnancy, and we assumed the same modelling framework for all outcomes.

We fit two-parent linear regression models for neonatal anthropometry, continuous cognitive score outcomes, and logistic regressions for binary outcomes representing preterm birth (less than 37 weeks’ gestation) or transfer to a neonatal special care unit (NSCU). The two-parent models include maternal cannabis and paternal cannabis. We compared the differences in the strength of the maternal cannabis and paternal cannabis associations with outcomes in unadjusted and adjusted models using Wald tests, with a null hypothesis of equal coefficients. Finally, we examined the percentage change in effect estimates for cannabis use between single and two-parent models.

## Results

Characteristics of the imputed and complete case samples were broadly similar, though the imputation recovered a greater representation of participants from lower SEP backgrounds. Here, we mainly discuss results from the imputed sample analyses, except to highlight specific differences with the complete case sample. Overall, 13.3% of fathers and 5.3% of mothers in the imputed sample used cannabis in some capacity before or during pregnancy, with 3.1% of mothers continuing into pregnancy (Table 2), figures higher than the complete case sample (Table S4). Among infants in the sample, the mean birth weight was 3374 g (SD 589), the mean birth length was 50.3 cm (SD 2.5), and the mean head circumference was 34.6 cm (SD 1.8). Across the imputed samples, 6.5% of women experienced a preterm birth, 11.6% of infants required admission to a neonatal special care nursery or unit (NSCU), and 0.9% of pregnancies resulted in perinatal death.

**Table 2:** Frequency and timing of cannabis use in pregnancy in ALSPAC from imputed sample

	Maternal		Paternal	
	No.	%	No.	%
Before and during pregnancy				
More than 1 time per week	169	1.1	721	4.8
One time per week	253	1.7	362	2.4
Less than 1 time per week	375	2.5	908	6.0
No use	14,216	94.7	13,022	86.7
Any cannabis use in pregnancy	472	3.1	1492	9.9
Total	15,013	100.0	15,013	100.0

Categorical variables for maternal and paternal cannabis before and during pregnancy: *more than 1 time per week* (uses more than once a week [> 1 ×/week], e.g., daily or several times a week); *1 time per week* (uses 1 time per week [1 ×/week]), and *less than 1 time per week (uses less than once a week [*< *1 ×/week]*, e.g., monthly or occasionally). Any cannabis use during pregnancy refers to any reported frequency of use during pregnancy only

Among mothers, 1.1% used cannabis more than once per week, 1.7% used cannabis 1 time per week, and 2.5% used cannabis less than once per week during preconception and pregnancy. Among the fathers, 4.8% used cannabis more than once per week, 2.4% used cannabis 1 time per week, and 6.1% used cannabis less than once per week before and during pregnancy. Among mothers using cannabis more than 1 time per week, 72% had partners with the same frequency of use, while 90% of mothers with no cannabis use also had partners who were non-users. The Pearson correlation coefficient between maternal and paternal cannabis use in pregnancy was 0.47 (p < 0.001).

### Socioeconomic patterning of cannabis use in ALSPAC

**Fig. 1:**
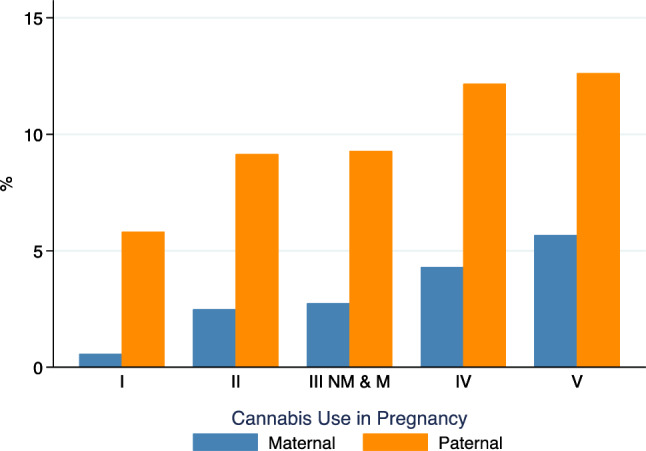
Frequency of maternal and paternal cannabis use in pregnancy by social class, ALSPAC imputed sample (n = 15,013). *Notes* social class refers to the highest parental socioeconomic position, categorized by occupational class (*I*  highest, *V*  lowest)

There was an inverse gradient between maternal and paternal cannabis use and SEP. In mothers, cannabis use frequency increased from 1.9% among those in the highest occupational social class group (I) to 8.4% among those in the lowest occupational social class group (V) and among fathers from 11.0 to 17.4% between groups I and V (Fig. 1). However, cannabis use prevalence showed less variation across levels of parental educational attainment, with a modest gradient between A level (equivalent to a year level higher than Canadian grade 12) and Certificate of Secondary Education (CSE) (increasing from 2.8 to 4.0% among mothers). Degree holders had a slightly higher prevalence at 3.3% (Fig. S2). Mothers who used cannabis were younger (mean age 25.8 vs. 28.1 years) and more likely to use tobacco (68.1% vs. 28.1%) or alcohol (30.9% vs. 17.8%) in the 1st trimester (Table 1). Although many cannabis users also smoked, 32% of mothers who reported cannabis use did not use tobacco. Finally, SEP defined by social class was strongly associated with study outcomes (Supplemental Figs. S3, S4).

### What is the association between cannabis use in pregnancy and neonatal anthropometry?

**Fig. 2:**
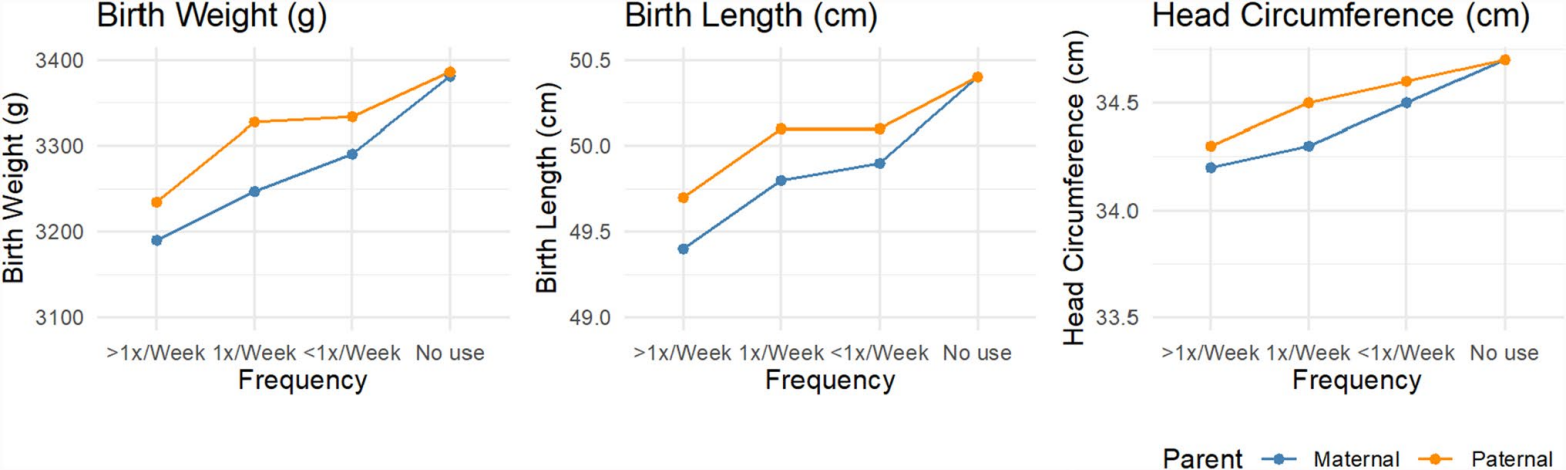
Mean neonatal anthropometry measures by maternal and paternal cannabis use in pregnancy, ALSPAC imputed sample

**Fig. 3:**
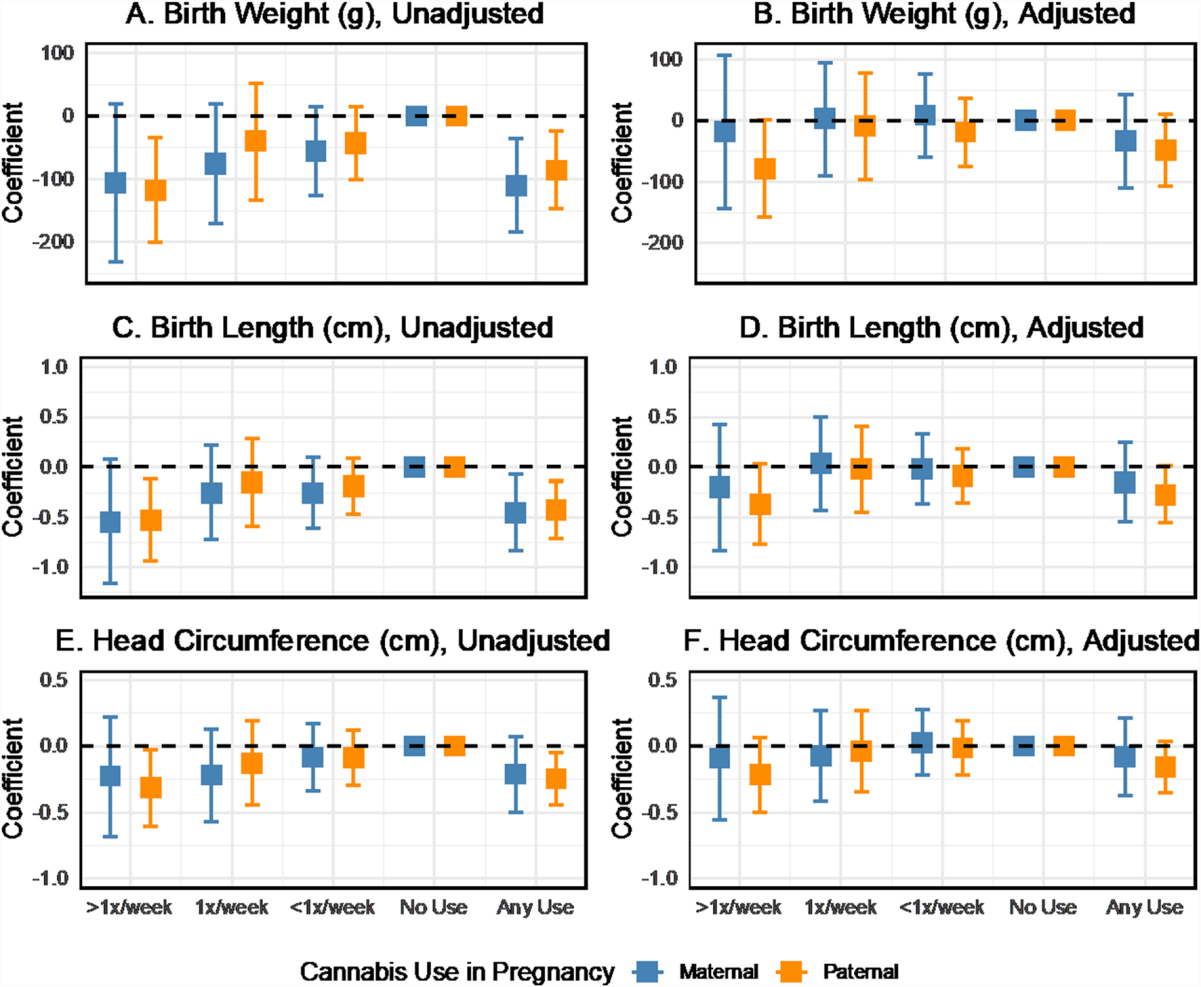
Coefficient estimates of the associations between maternal and paternal cannabis use and neonatal anthropometry, with and without adjustment for covariates, two-parent models, ALSPAC imputed sample, data provided in Supplemental Table S5

Mean neonatal anthropometry varied by maternal and paternal cannabis use during pregnancy (Fig. 2). Increased frequency of cannabis consumption by mothers and partners was associated with a trend toward lower birth weight and a smaller birth length in unadjusted analyses. Two-parent regression models also showed inverse associations between the frequency of cannabis use in pregnancy and neonatal anthropometry (Fig. 3). However, estimates for specific use categories were too imprecise to detect effects reliably. The beta coefficients for birth weight were − 110.2 (95% CI − 185.1 to − 35.3) for any maternal cannabis use in pregnancy and − 85.1 (95% CI − 145.7 to − 24.6) for any paternal use in pregnancy, with no difference detected in the magnitude of maternal and paternal effects. The birth length and head circumference results followed a similar pattern in the unadjusted models. In adjusted models, the magnitude of the cannabis use associations with neonatal anthropometry was consistent but estimated with reduced precision. For example, the coefficients for birthweight were − 35.0 (95% CI − 111.4 to 41.4) for maternal use and − 48.5 (95% CI − 106.6 to 9.6) for paternal use. We also fit models based on the non-missing complete case sample (Fig. S5). These results show some sign changes for maternal cannabis use in adjusted models, which may be related to the influence of missing data on the estimates.

### What is the association between cannabis use in pregnancy and perinatal outcomes?

**Fig. 4:**
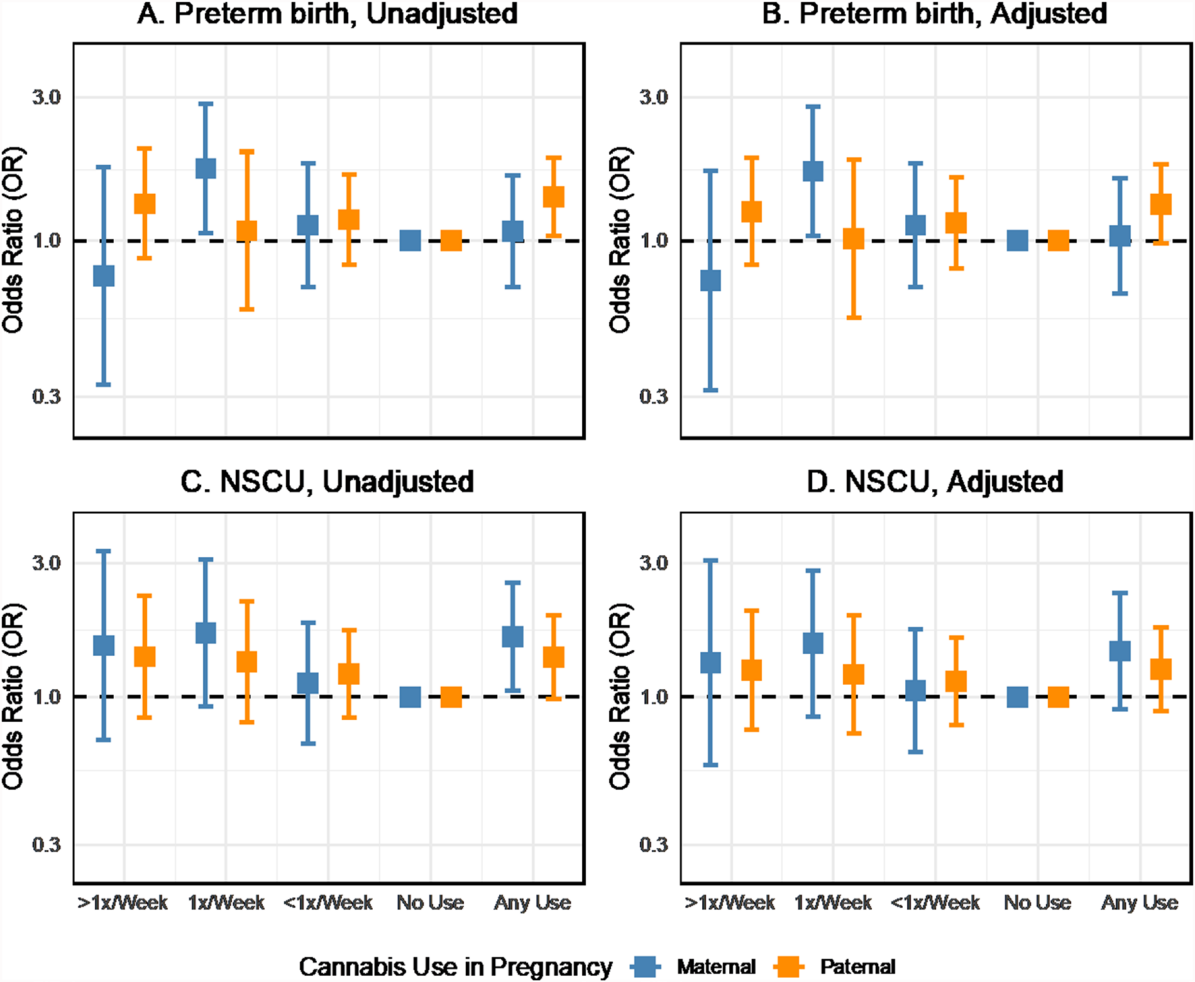
Odds ratios for the associations between maternal and paternal cannabis use and perinatal outcomes, with and without adjustment for covariates, two-parent models, ALSPAC imputed sample, data provided in Supplemental Table S6. *GCSE* General Certificate of Secondary Education Scores, *SDQ* Strengths and Difficulties Questionnaire (Hyperactivity Score), *IQ* Intelligence Quotient

In logistic regressions of perinatal outcomes on maternal and paternal cannabis use in pregnancy unadjusted for other covariates, cannabis use was generally associated with increased odds of adverse obstetrical outcomes such as preterm birth. However, there was some variation in the direction and magnitude of the association across cannabis use frequency (Fig. 4A). Maternal use 1 time per week was associated with increased odds of preterm birth (OR 1.75, 95% CI 1.06 to 2.88) in the two-parent unadjusted model. However, the association between any maternal cannabis use and preterm birth was weak and imprecisely estimated (OR 1.08, 95% CI 0.70 to 1.66). Any paternal cannabis use in pregnancy was associated with preterm birth (OR 1.40, 95% CI 1.04 to 1.90). We found no evidence to reject the null hypothesis of no difference in the association for any maternal or paternal cannabis use in pregnancy (p = 0.41). Additional adjustment for potential confounders moderately attenuated the associations between cannabis use and preterm birth, although the estimate for maternal weekly use remained similar (OR 1.71, 95% CI 1.04 to 2.82, Fig. 4B). The direction and magnitude of the cannabis-preterm birth associations were highly consistent between the two-parent unadjusted and adjusted models in the imputed sample. However, more variability was seen in the complete case analysis (Fig. S6).

The point estimates for the association between cannabis use in pregnancy and newborns requiring NSCU admission were positive but with uncertainty for the frequency of use categories (Fig. 4C). Any maternal cannabis use in pregnancy was associated with increased odds of NSCU admission (OR 1.64, 95% CI 1.05 to 2.56), as was any paternal cannabis (OR 1.39, 95% CI 0.99 to 1.95) in the two-parent model (p-value for maternal–paternal difference: 0.63). Including additional covariates in the models attenuated the maternal and paternal associations by about 30% (from 1.64 to 1.46 and 1.39 to 1.26). Still, the effect sizes were similar between the unadjusted and adjusted two-parent models (Fig. 4D).

### What is the association between maternal cannabis use and cognitive outcomes?

**Fig. 5:**
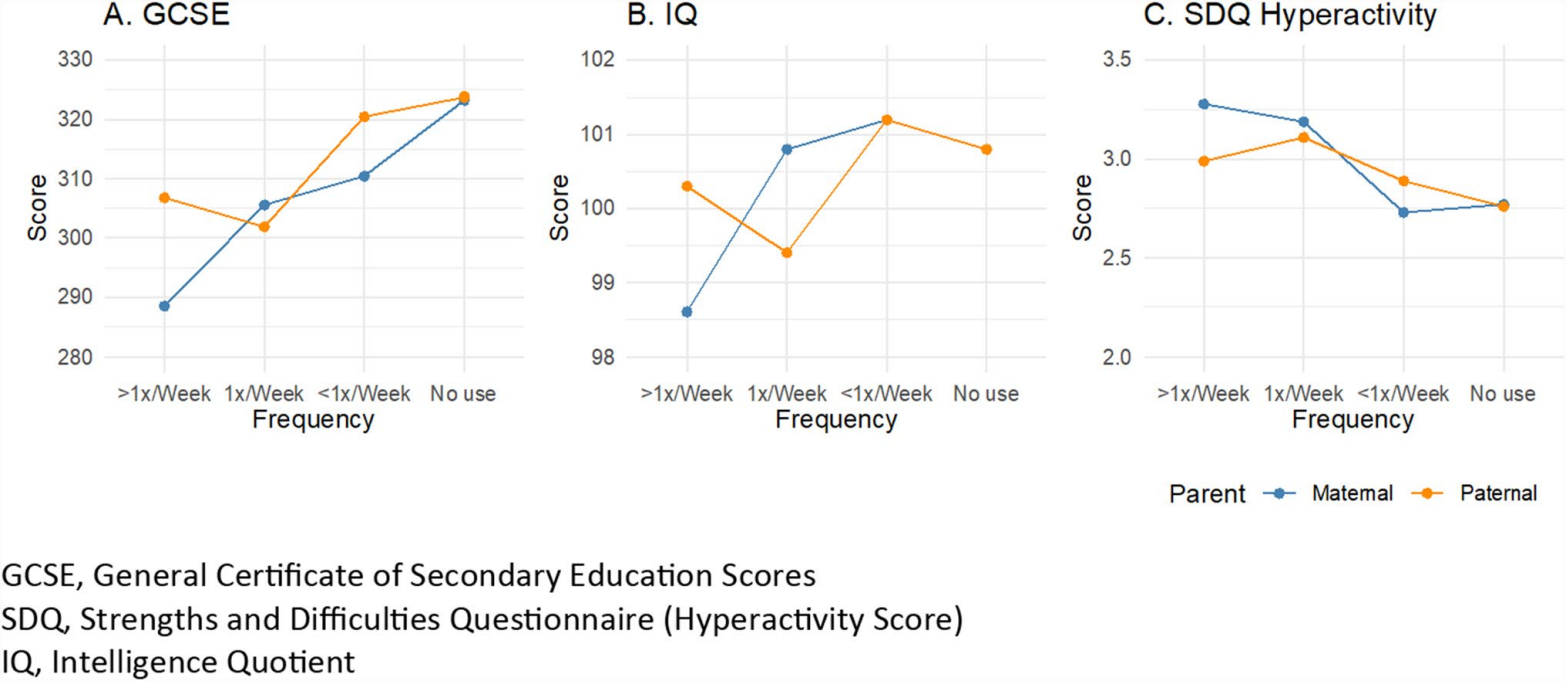
Mean offspring GCSE, IQ, and SDQ hyperactivity scores by maternal and paternal cannabis use in pregnancy, ALSPAC imputed sample

**Fig. 6:**
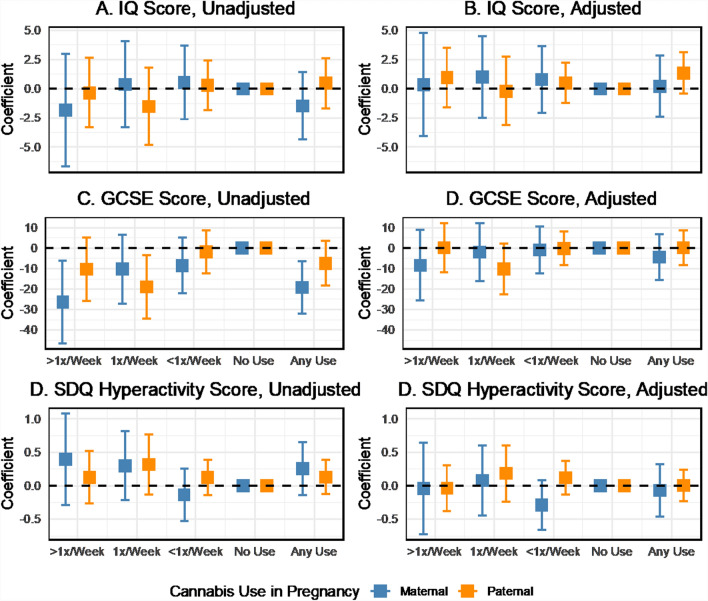
Coefficient estimates of the associations between maternal and paternal cannabis use and cognitive outcomes, with and without adjustment for covariates, two-parent models, ALSPAC imputed sample, data provided in Supplemental Table S7

Descriptively, educational scores at age 16 (GCSE) were inversely correlated with cannabis use during pregnancy, particularly maternal use (Fig. 5A). IQ scores at age 8 did not show a graded association with cannabis use (Fig. 5B) and were less consistent than other outcomes, but increases in hyperactivity scores from the SDQ were found with increasing maternal and paternal cannabis use (Fig. 5C). Two-parent regression analyses revealed no substantial associations between maternal or paternal cannabis use in pregnancy and IQ scores at age 8 unadjusted or adjusted for confounders (Figs. 6A, B). Maternal cannabis use more than once per week was associated with lower GCSE scores among offspring at age 16 (b = − 26.4, 95% CI − 46.6 to − 6.2), as was any maternal cannabis use in pregnancy (b = − 19.2, 95% CI − 32.0 to − 6.3) in the unadjusted two-parent regression (Fig. 6C). Paternal weekly cannabis consumption was also inversely associated with GCSE scores (b = − 18.9, 95% CI − 34.5 to − 3.4). These associations were attenuated by up to 77% toward the null after including the additional covariates in the adjusted model (Fig. 9D). Maternal cannabis use at least once or more than once per week was positively associated with increased SDQ scores for hyperactivity in the unadjusted analysis (Fig. 6E). However, the confidence intervals indicated uncertainty in the estimates. Paternal cannabis also showed positive associations with hyperactivity, with similar levels of uncertainty. Wald tests indicated no apparent differences between the maternal and paternal coefficients for cannabis across all cognitive outcomes.

### Comparison of maternal cannabis associations in maternal-only versus two-parent models

**Fig. 7:**
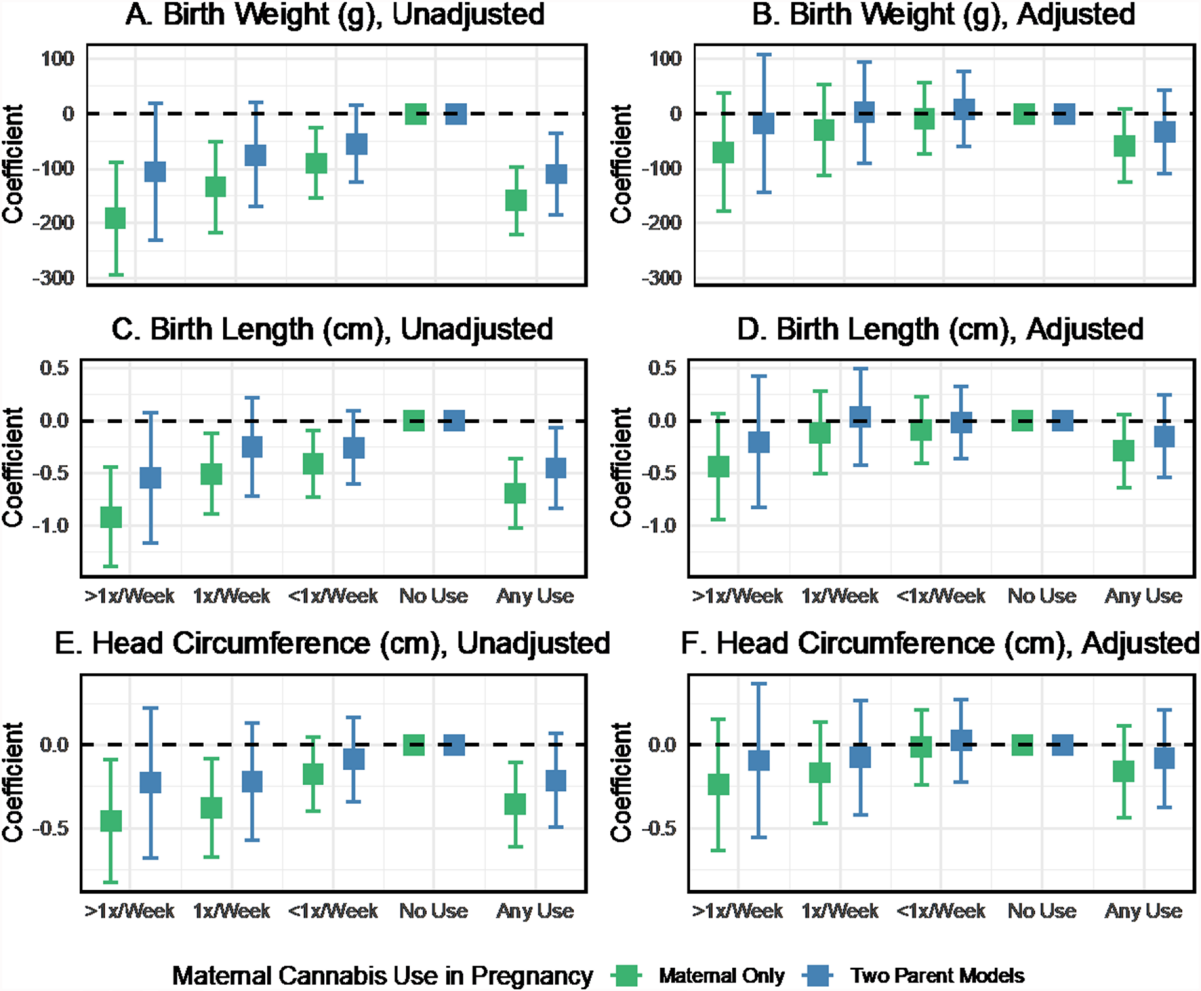
Comparison of coefficient estimates of the associations between maternal cannabis use and neonatal anthropometry, with and without adjustment for covariates, two-parent models vs. Maternal-only models, ALSPAC imputed sample

**Fig. 8:**
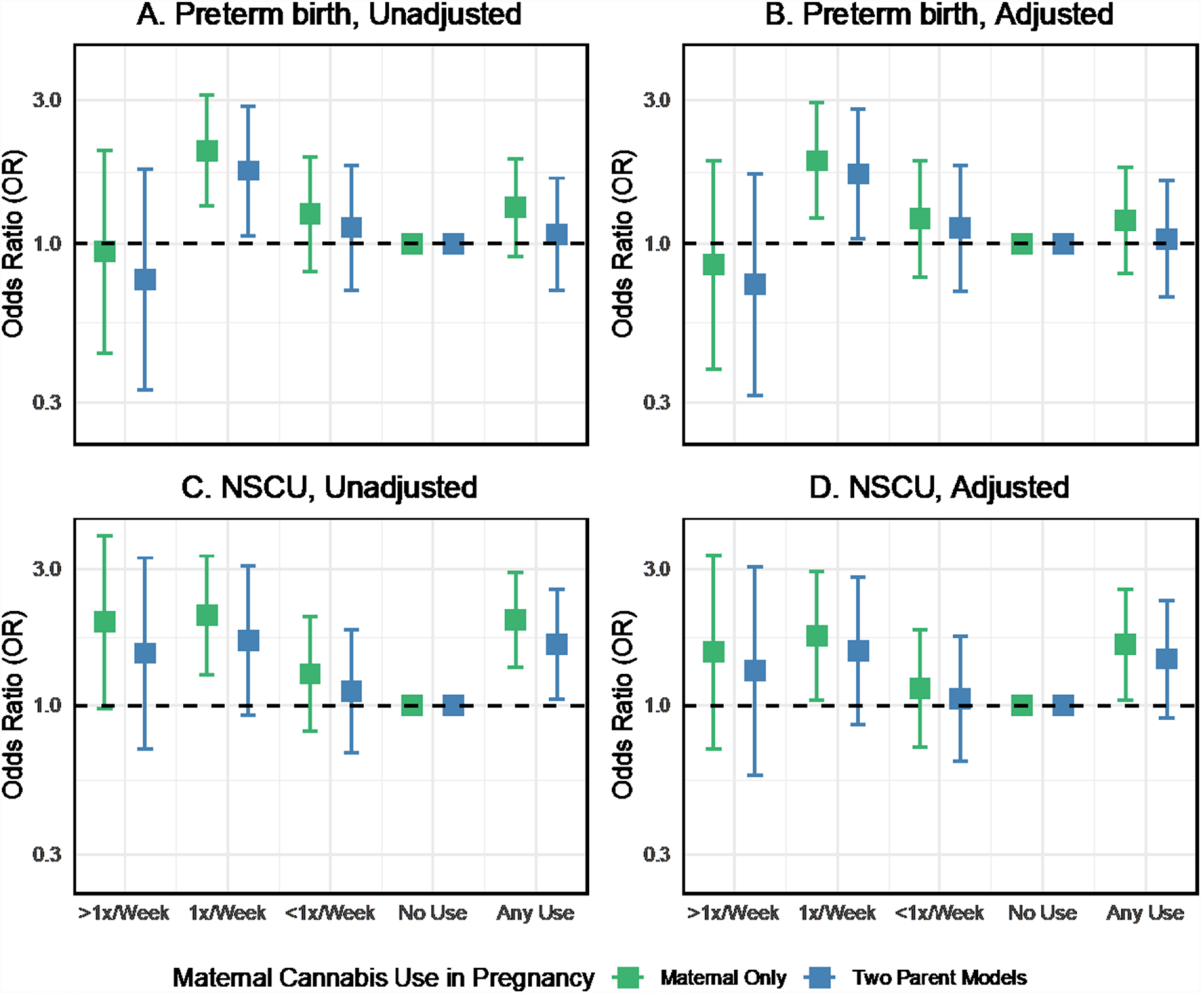
Comparison of odds ratios of the associations between maternal cannabis use and obstetrical outcomes, with and without adjustment for covariates: two-parent models vs. maternal-only models, ALSPAC imputed sample

**Fig. 9:**
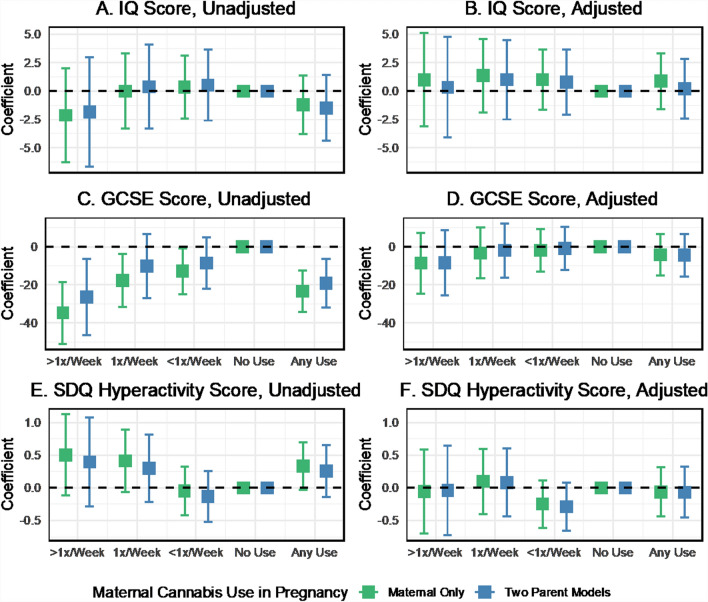
Comparison of coefficient estimates of the associations between maternal cannabis use and cognitive outcomes, with and without adjustment for covariates: two-parent models vs. maternal-only models, ALSPAC imputed sample

In the unadjusted maternal-only regression models for neonatal anthropometry, the effect estimates for cannabis use were generally about 30–50% stronger than the two-parent unadjusted models (Fig. 7**, **Panels A, C, E). Covariate adjustment attenuated the cannabis associations in single-parent models, although point estimates remained negative, suggesting an inverse association (Fig. 7**, **Panels B, D, F). A similar pattern was seen for cannabis associations with preterm birth and NSCU admission (Fig. 8) and cognitive and neurodevelopmental outcomes (Fig. 9). In maternal-only adjusted models, cannabis use frequency of 1 time per week was associated with increased odds of preterm birth (OR 1.89, 95% CI 1.21 to 2.95) and NSCU admission (OR 1.75, 95% CI 1.04 to 2.94) and any cannabis use was also associated with an increased odds of NSCU admission (OR 1.64, 95% CI 1.05 to 2.56).

## Discussion

In this study, we had four principal findings. First, we found evidence of unadjusted associations between maternal cannabis use in pregnancy and neonatal anthropometry, perinatal outcomes and offspring cognitive development. Associations were, however, attenuated in covariate-adjusted models that included household-level factors such as SEP, implying the presence of confounding. Second, although we found evidence of an association between maternal cannabis use in pregnancy and outcomes, these associations were generally similar to those for paternal cannabis use, our negative control, with no strong evidence against the null hypothesis of concordant maternal and paternal cannabis use associations with outcomes. Although we are unable to definitively conclude that there is no difference in maternal and paternal effects, which may exist in other larger datasets, the ALSPAC data suggest that household-level factors, including socioeconomic conditions, may be driving cannabis–outcome associations, diminishing the likelihood of maternal-specific biological or intrauterine mechanisms. Third, the maternal-only models of cannabis use on outcomes were markedly attenuated by including covariates and household-level SEP markers, again strongly implying the presence of confounding. Last, controlling for covariates did not wholly eliminate all associations between cannabis use and outcomes, suggesting a residual association after covariate adjustment or that there may exist other important but unaccounted-for family-level or environmental factors.

### Plausibility of association

We found some evidence of a dose–response association between the frequency of maternal cannabis consumption in pregnancy and outcomes, but this was limited to maternal-only models without paternal use or covariate adjustment. Additional support for potential biological associations between cannabis use and offspring outcomes is available from animal models [30, 31]. For example, a study of rats gestationally exposed to a synthetic cannabinoid agonist demonstrated increased hyperactivity and memory impairment in offspring compared to controls [32]. Other animal models of Δ^9^-THC and synthetic cannabinoid exposure in pregnancy suggest similar cognitive impairments in the offspring [33]. Although useful, it's not clear how well animal studies can represent Δ^9^-THC usage patterns and absorption in human populations.

Cannabinoids can cross the placenta and interact with the fetus [9]. Therefore, there is biological plausibility to the hypothesis that fetal exposure to cannabis may in some way alter fetal development. However, epidemiological investigations into this question are limited primarily to observational designs. Several international investigations into the question have yielded inconsistent results, likely due to methodological differences across studies and varying strategies for dealing with confounding [3, 34–40]. A previous study using the paternal control approach in Generation R came to a similar conclusion that cannabis associations with behavioural problems may be generated through residual confounding [19]. Cannabis use in many high-income populations highly correlates with confounding factors such as SEP [11]. Further, SEP is also strongly related to birth outcomes, meaning that any observational study of cannabis use and outcomes may produce spurious associations depending on the SEP indicators available and statistical adjustment strategy. In addition, removing residual confounding by SEP in specific observational datasets may be impossible even with multivariable adjustment [41].

Here we discuss the limitations of our study, data, and analytical approach. Launched in 1991, ALSPAC was conducted when herbal cannabis products were far less potent than currently available. For instance, the concentrations of Δ^9^-THC in cannabis plant material in the United States have risen from 4% in 1995 to about 14% in 2019 [10], mirroring potency assessments of cannabis in European countries [42]. Data on other forms of cannabis, including edibles, which have grown in popularity following legalization [43], were not available in ALSPAC. The availability of cannabis products with limited Δ^9^-THC potency at the time of ALSPAC recruitment may be related to finding less robust maternal-specific associations between cannabis and pregnancy outcomes and offspring cognitive development. In addition, ALSPAC collected data related to prenatal exposures via postal questionnaires, leading to missing data on maternal and paternal cannabis use and substance use. The problem was particularly substantial for paternal cannabis use, where about 35% of the cohort had missing data; thus, our approach here was to use multiple imputation to recover these missing observations and to reduce potential biases arising from a complete-case analysis. While we included a range of covariates and auxiliary variables within the multiple imputation model to capture all factors potentially predictive of missing data [44], it is possible that assumptions with the imputation models were not fully met, which could introduce bias. In addition, even with our approach of 50 imputed datasets, some uncertainty remains in the effect estimates. Although we did not explicitly consider the impacts of whether the partners were biological fathers, two points merit consideration. First, using non-biological partners or father figures as negative controls would not appear to compromise the validity of the negative control design as the father's impact must be environmental, which would be equal for biological and non-biological fathers. Second, in ALSPAC, mothers invited the individual they felt was most appropriate to complete the partner questionnaire [45], and 95% reported that individual as the biological father. Prior studies assumed that up to 10% may be non-biological [23], and non-paternity would only influence heritable components in covariates and outcomes in the fraction of the sample with non-biological fathers. A small proportion (~ 6%) of the ALSPAC participants were recruited after age 7 who had been born in the study area but were not initially recruited. Cannabis exposure for this group was handled by multiple imputation, which may introduce some noise and reduce the precision of these estimates, but we excluded all retrospective reporting. Finally, although many statistical tests were performed, we did not adjust for multiple comparisons [46]. Adjusting for multiple comparisons would reduce the likelihood of false positives but can also lead to more false negatives. Given that our main analyses involved maternal–paternal comparisons, the concern would be related to false negative findings or no maternal–paternal difference, which may underestimate the maternal-specific effect of cannabis. In addition, we focused the statistical testing only on the overall maternal–paternal comparisons, which reduces the total number of comparisons and reliance on statistical testing.

ALSPAC has two unique features to investigate the associations between maternal cannabis use and offspring outcomes. First, ALSPAC collected a rich set of SEP markers, including maternal and paternal education, social class, and CAMSIS score, covariates unavailable in other clinical datasets or registries [11]. Although other factors are available, the broader SEP measures, particularly the CAMSIS indicator, robustly capture many dimensions of SEP. Additional adjustment for variables related to SEP may further reduce bias, given that there may be an imperfect capture of SEP with our selected variables. However, maternal cannabis and paternal cannabis were fully attenuated in adjusted models, indicating a high likelihood of shared confounding, and the association would be unlikely to change substantially by adding additional control variables. Second, ALSPAC has data on paternal cannabis use during pregnancy, which is the ideal negative control exposure to test a potential direct biological effect of maternal cannabis use on perinatal and offspring outcomes [20]. Using these features, we demonstrated that the associations between maternal cannabis and outcomes were attenuated by adjusting for confounding factors, including SEP. In addition, none of the associations between maternal cannabis use were quantitatively more substantial than the paternal negative control exposure. Our findings revealed a considerable overlap between the adjusted maternal and paternal estimates, which may be partly due to the increased noise of additional adjustment. However, as a negative control, we would expect paternal cannabis use to have no or limited association with outcomes assuming a maternal-specific intrauterine effect. Instead, these data suggest a robust SEP patterning of the exposure–outcome relationship, not a maternal-specific effect of cannabis.

### Concluding remarks

Our findings strongly suggest that associations between maternal cannabis use in pregnancy and adverse perinatal or cognitive outcomes in offspring may be driven by socioeconomic confounding. However, maternal cannabis use in pregnancy remains a marker of potentially high-risk pregnancy outcomes and of longer-term outcomes in offspring, which may be associated with SEP. Other research examining maternal cannabis-related associations should consider a similar negative control design.

## Supplementary Information

Below is the link to the electronic supplementary material.

Supplementary file1 (DOCX 658 kb)
