# Supplementary material for: Maternal cannabis use in pregnancy, perinatal outcomes, and cognitive development in offspring: a longitudinal analysis of the ALSPAC cohort using paternal cannabis use as a negative control exposure
Source: Eur J Epidemiol. 2025 May 12;40(5):549–62. doi: 10.1007/s10654-025-01233-w (PMC12170737; doi:10.1007/s10654-025-01233-w)
Supplement: Supplementary file 1 — Supplementary file1 (DOCX 658 kb) [file 10654_2025_1233_MOESM1_ESM.docx]

**SUPPLEMENTAL APPENDIX**

## **Multiple imputation**

Multiple Imputation by Chained Equations (MICE) (Royston & White, 2011) was conducted to impute missing data from ALSPAC. From the 15,645 children enrolled in ALSPAC, only 3,905 had complete data on outcomes, exposures and all covariates (**Figure S1**). Data were imputed for all variables except for sex and month of birth, resulting in an imputed sample size of 15,013. We imputed 50 datasets for analysis. **Table S1** shows the variables used in the analyses and the extent of missingness across these variables. Due to patterns of missingness across the variables of interest, only 26% of ALSPAC participants with sex and month of birth had complete data on all other variables, with item missingness increasing broadly linearly (**Table S2**). Some auxiliary/supplementary variables were used to improve the accuracy of the imputation models (**Table S3**). Some of these were selected from the National Pupil Database, given the high accuracy and completeness of this data relative to the number of participants with educational linkage data available.

## **Supplementary Figure S1** STROBE diagram of attrition in the complete case sample.

Sex and month of birth data available (n = 15,013)

Maternal cannabis available

(n = 12,553)

Missing paternal cannabis

(n = 3,339)

Missing sex and month of birth data (n = 632)

Missing maternal cannabis

(n = 2,460)

Children enrolled in ALSPAC (n = 15,645)

Maternal and paternal cannabis available (n = 9,214)

Missing socioeconomic position

(n = 1,291)

Family socioeconomic position available (n = 7,923)

Missing perinatal outcomes

(n = 3,066)

Perinatal outcomes data available (n = 4,857)

(n = 4,857)

Missing covariates

(n = 952)

Complete cases

(n = 3,905)

## **Supplementary Table S1** Main variables, the extent of overall missingness, and regression commands used for multiple imputation in the ALSPAC sample from the 15,013 participants who had data on sex at birth and month of birth

| Variable | Responses | Missingness (%) | Regression command |
| --- | --- | --- | --- |
| Sex | 15,013 | 0 | [No missing data in sample] |
| Preterm birth | 14,130 | 5.88 | [from gestational age- Linear] |
| Maternal age | 14,064 | 6.32 | Linear |
| Birthweight | 13,883 | 7.53 | Linear |
| Maternal smoking | 13,158 | 12.36 | Ordered Logistic |
| Maternal alcohol use | 13,150 | 12.41 | Ordered Logistic |
| Maternal drug use | 13,018 | 13.29 | Ordered Logistic |
| Parity | 12,855 | 14.37 | Linear |
| Maternal cannabis use after first trimester | 12,555 | 16.37 | Ordered Logistic |
| Maternal cannabis use in first trimester | 12,553 | 16.39 | Ordered Logistic |
| Maternal cannabis use 6 before pregnancy | 12,544 | 16.45 | Ordered Logistic |
| Parental education | 12,493 | 16.79 | Ordered Logistic |
| BMI | 11,605 | 22.7 | Linear |
| Parental SEP | 11,063 | 26.31 | Ordered Logistic |
| GCSE points | 10,842 | 27.78 | Linear |
| Paternal alcohol use | 9,924 | 33.9 | Ordered Logistic |
| Paternal cannabis use before pregnancy | 9,747 | 35.08 | Ordered Logistic |
| Paternal cannabis use after pregnancy | 9,668 | 35.6 | Ordered Logistic |
| Paternal drug use | 9,620 | 35.92 | Ordered Logistic |
| Paternal smoking | 9,479 | 36.86 | Ordered Logistic |
| Birth length | 8,483 | 43.5 | Linear |
| NCSU | 7,962 | 46.97 | Multinomial logistic |
| IQ | 7,338 | 51.12 | Linear |

## **Supplementary Table S2** Patterns of item missingness in the ALSPAC sample from the 15,013 participants who had data on sex at birth and month of birth

| Number of missing items | n | % |
| --- | --- | --- |
| 0 | 1,309 | 8.72 |
| 1 | 2,376 | 15.83 |
| 2 | 2,188 | 14.57 |
| 3 | 1,626 | 10.83 |
| 4 | 1,124 | 7.49 |
| 5 | 907 | 6.04 |
| 6 | 940 | 6.26 |
| 7 | 924 | 6.15 |
| 8 | 673 | 4.48 |
| 9 | 487 | 3.24 |
| 10 | 312 | 2.08 |
| 11 | 163 | 1.09 |
| 12 | 112 | 0.75 |
| 13 | 125 | 0.83 |
| 14 | 80 | 0.53 |
| 15 | 136 | 0.91 |
| 16 | 161 | 1.07 |
| 17 | 159 | 1.06 |
| 18 | 130 | 0.87 |
| 19 | 95 | 0.63 |
| 20 | 65 | 0.43 |
| 21 | 252 | 1.68 |
| 22 | 540 | 3.6 |
| 23 | 129 | 0.86 |

## **Supplementary Table S3** Supplementary variables used in imputation.

| Variable | Justification for inclusion |
| --- | --- |
| Month of delivery | Children born earlier in the academic year tend to perform better in educational tests |
| Academic Year* | To account for any period effects in educational test performance |
| Does pupil have Special Educational Need* | Children with Special Educational Needs tend to score lower in educational tests than those without Special Educational Needs |
| Whether pupil is known to be eligible for Free School Meals* | Eligibility for Free School Meals is a proxy for low-income |
| IQ at age 15 | IQ correlates highly between ages 8 and 15 |

* denotes variables taken from the National Pupil Database administrative education dataset.

**Supplementary Table S4** Frequency and Timing of Cannabis Use in Pregnancy in ALSPAC from complete case sample

|  | **Maternal** | |  | **Paternal** | |
| --- | --- | --- | --- | --- | --- |
|  | **No.** | **%** |  | **No.** | **%** |
| Before and during pregnancy |  |  |  |  |  |
| At least weekly | 31 | 0.8 |  | 110 | 2.8 |
| Weekly | 52 | 1.3 |  | 39 | 1.0 |
| Less than weekly | 74 | 1.9 |  | 117 | 3.0 |
| Did not use | 3,748 | 96.0 |  | 3,639 | 93.2 |
| Any Cannabis Use Before and in Pregnancy | 157 | 4.0 |  | 266 | 6.8 |
| Any Cannabis Use in Pregnancy | 77 | 2.0 |  | 205 | 5.2 |
| Total | 3,905 | 100.0 |  | 3,905 | 100.0 |

Categorical variables for maternal and paternal cannabis before and during pregnancy: *more than one time per week* (uses more than once a week [>1x/week], e.g., daily or several times a week); *one time per week* (uses one time per week [1x/week]), and *less than one time per week (uses less than once a week [<1x/week]*, e.g., monthly or occasionally). Any cannabis use during pregnancy refers to any reported frequency of use during pregnancy only.

## **References**

Royston, P., & White, I. (2011). Multiple Imputation by Chained Equations (MICE): Implementation in Stata. *Journal of Statistical Software*. https://doi.org/10.18637/jss.v045.i04

**Figure S2** Frequency of Maternal and Paternal Cannabis Use in Pregnancy by Educational Attainment, ALSPAC, Imputed Sample

Educational attainment refers to the highest level of parental education. CSE stands for Certificate of Secondary Education; Degree represents the highest level of educational attainment.

**Figure S3** Mean Neonatal Anthropometry Measurements by Social Class, ALSPAC

Note: Social Class is the highest parental socioeconomic position.

**Figure S4** Prevalence of Preterm Birth and Admission to Neonatal Special Care Unit (NSCU) by Social Class, ALSPAC

Note: Social Class is the highest parental socioeconomic position.


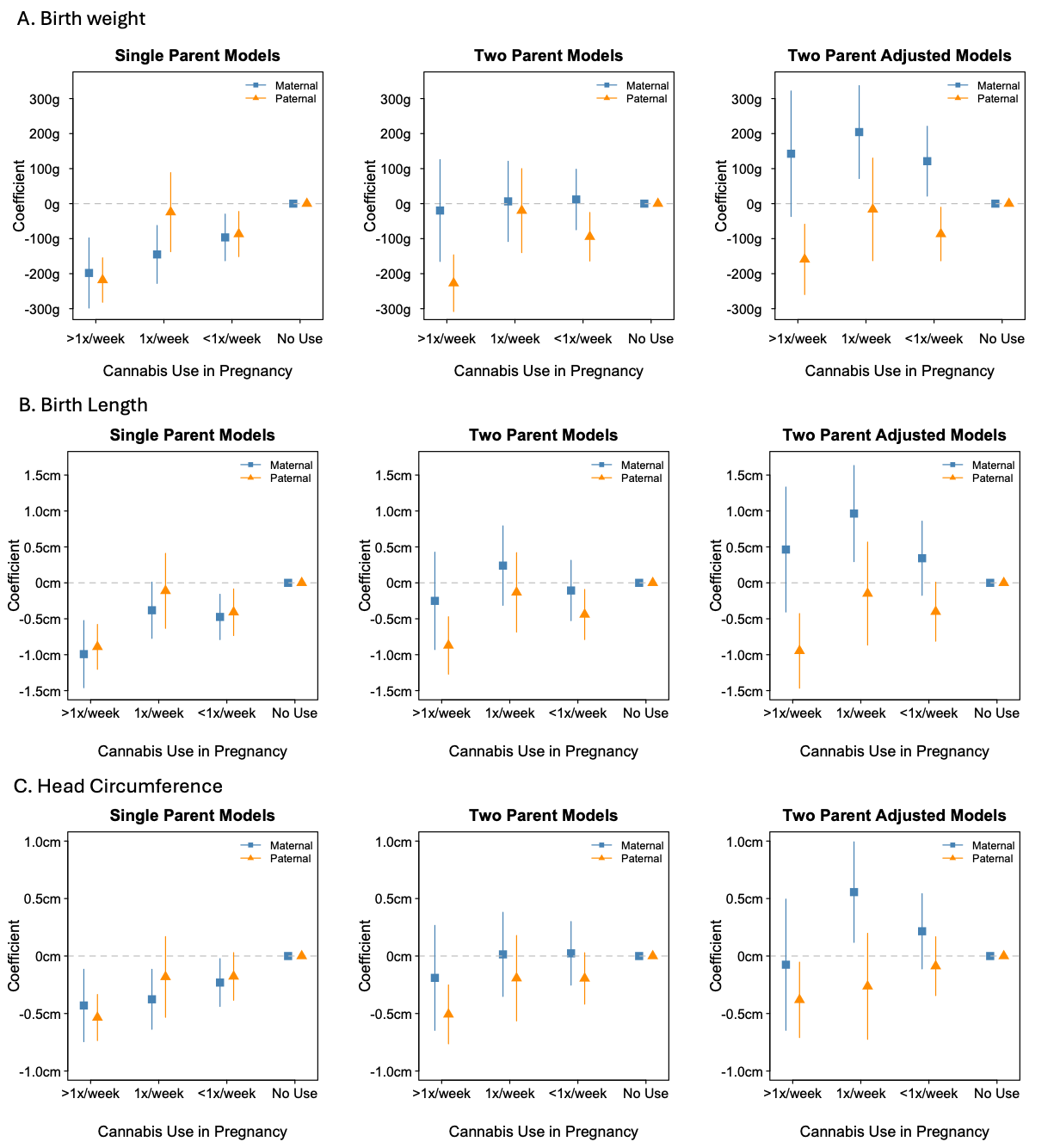


**Figure S5** Coefficient Estimates of the Association Between Maternal and Paternal Cannabis Use and Neonatal Anthropometry under Different Model Specifications In The Complete Case Sample, ALSPAC

Models are based on the non-missing complete case sample, with additional list-wise deletion performed when the full set of covariates is added (right column). Note the sign change for maternal cannabis use in adjusted models, suggesting that excluding a non-random subset of the cohort due to missing data may bias the estimates.


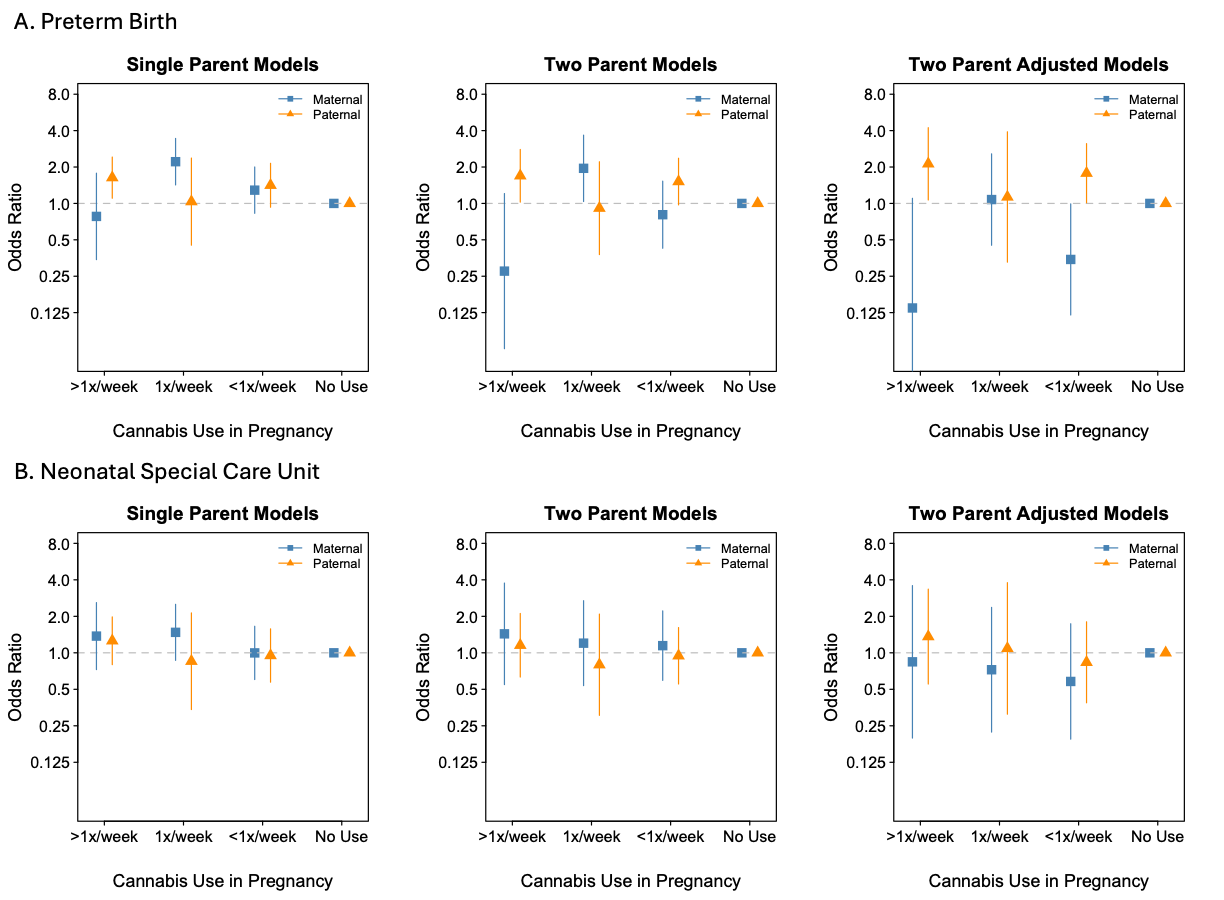


**Figure S6** Odds Ratios for the Associations Between Maternal and Paternal Cannabis Use and Perinatal Outcomes Under Different Model Specifications in the Complete Case Sample, ALSPAC

Models are based on the non-missing complete case sample, with additional list-wise deletion performed when the full set of covariates is added (right column). Note the fluctuation in maternal cannabis use estimates in adjusted models, suggesting that excluding a non-random subset of the cohort due to missing data may bias the estimates.

**Supplementary Table S5** Coefficient Estimates of the Associations between Maternal and Paternal Cannabis Use and Neonatal Anthropometry, with and without adjustment for covariates, two-parent models, ALSPAC imputed sample

|  | Birth weight | | | |  | Birth length | | | |  | Head Circumference | | | |
| --- | --- | --- | --- | --- | --- | --- | --- | --- | --- | --- | --- | --- | --- | --- |
|  | Unadjusted | | Adjusted | |  | Unadjusted | | Adjusted | |  | Unadjusted | | Adjusted | |
| Maternal Cannabis | b | 95% CI | b | 95% CI |  | b | 95% CI | b | 95% CI |  | b | 95% CI | b | 95% CI |
| >1x/Week | -105.94 | (-231.11,19.22) | -18.24 | (-143.81,107.33) |  | -0.54 | (-1.17,0.08) | -0.20 | (-0.83,0.43) |  | -0.23 | (-0.68,0.22) | -0.09 | (-0.56,0.37) |
| 1x/Week Weekly | -75.14 | (-170.33,20.06) | 2.77 | (-89.23,94.77) |  | -0.25 | (-0.72,0.22) | 0.04 | (-0.43,0.50) |  | -0.22 | (-0.57,0.13) | -0.08 | (-0.42,0.27) |
| <1x/Week | -55.36 | (-125.87,15.15) | 8.50 | (-59.28,76.27) |  | -0.26 | (-0.61,0.09) | -0.02 | (-0.36,0.33) |  | -0.09 | (-0.34,0.17) | 0.03 | (-0.22,0.27) |
| No use | 0.00 |  | 0.00 |  |  | 0.00 |  | 0.00 |  |  | 0.00 |  | 0.00 |  |
| Any Use in Pregnancy | -110.16 | (-185.05,-35.28) | -33.25 | (-109.66,43.17) |  | -0.45 | (-0.84,-0.07) | -0.15 | (-0.54,0.25) |  | -0.21 | (-0.49,0.07) | -0.08 | (-0.37,0.21) |
| Paternal Cannabis |  |  |  |  |  |  |  |  |  |  |  |  |  |  |
| >1x/Week | -117.74 | (-200.27,-35.68) | -78.59 | (-158.11,0.53) |  | -0.52 | (-0.93,-0.12) | -0.36 | (-0.76,0.03) |  | -0.31 | (-0.60,-0.03) | -0.22 | (-0.50,0.06) |
| 1x/Week Weekly | -39.72 | (-132.43,52.61) | -8.67 | (-95.52,77.90) |  | -0.15 | (-0.59,0.29) | -0.02 | (-0.44,0.41) |  | -0.13 | (-0.45,0.19) | -0.04 | (-0.35,0.27) |
| <1x/Week | -42.67 | (-99.95,14.32) | -19.17 | (-74.17,35.57) |  | -0.19 | (-0.47,0.09) | -0.09 | (-0.36,0.18) |  | -0.09 | (-0.30,0.12) | -0.01 | (-0.22,0.19) |
| No Use | 0.00 |  | 0.00 |  |  | 0.00 |  | 0.00 |  |  | 0.00 |  | 0.00 |  |
| Any Use in Pregnancy | -85.14 | (-145.68,-24.60) | -48.26 | (-106.30,9.79) |  | -0.42 | (-0.71,-0.13) | -0.27 | (-0.55,0.01) |  | -0.25 | (-0.44,-0.05) | -0.16 | (-0.35,0.04) |
| Wald Tests of  Maternal-Paternal |  |  |  |  |  |  |  |  |  |  |  |  |  |  |
| >1x/Week | 0.81 |  | 0.58 |  |  | 0.80 |  | 0.78 |  |  | 1.00 |  | 0.83 |  |
| 1x/Week Weekly | 0.65 |  | 0.88 |  |  | 0.80 |  | 0.88 |  |  | 0.75 |  | 0.90 |  |
| <1x/Week | 0.90 |  | 0.51 |  |  | 0.97 |  | 0.73 |  |  | 0.79 |  | 0.70 |  |
| Any Use in Pregnancy | 0.68 |  | 0.78 |  |  | 0.92 |  | 0.68 |  |  | 0.86 |  | 0.70 |  |

**Supplementary Table S6** Odds Ratios for the Associations between Maternal and Paternal Cannabis Use and Perinatal Outcomes, with and without adjustment for covariates, two-parent models, ALSPAC imputed sample

|  | Preterm Birth | | | |  | Neonatal Special Care Admission | | | |
| --- | --- | --- | --- | --- | --- | --- | --- | --- | --- |
|  | Unadjusted | | Adjusted | |  | Unadjusted | | Adjusted | |
| Maternal Cannabis | OR | 95% CI | OR | 95% CI |  | OR | 95% CI | OR | 95% CI |
| >1x/Week | 0.76 | (0.33,1.78) | 0.74 | (0.31,1.73) |  | 1.53 | (0.71,3.30) | 1.32 | (0.57,3.06) |
| 1x/Week Weekly | 1.75 | (1.06,2.88) | 1.71 | (1.04,2.82) |  | 1.69 | (0.93,3.09) | 1.55 | (0.86,2.82) |
| <1x/Week | 1.13 | (0.70,1.83) | 1.13 | (0.70,1.83) |  | 1.12 | (0.68,1.85) | 1.06 | (0.64,1.75) |
| No use | 1.00 |  | 1.00 |  |  | 1.00 |  | 1.00 |  |
| Any Use in Pregnancy | 1.08 | (0.70,1.66) | 1.04 | (0.66,1.62) |  | 1.64 | (1.05,2.56) | 1.46 | (0.91,2.35) |
| Paternal Cannabis |  |  |  |  |  |  |  |  |  |
| >1x/Week | 1.33 | (0.87,2.03) | 1.25 | (0.83,1.90) |  | 1.39 | (0.85,2.29) | 1.25 | (0.77,2.04) |
| 1x/Week Weekly | 1.08 | (0.59,1.99) | 1.02 | (0.55,1.88) |  | 1.33 | (0.81,2.19) | 1.21 | (0.74,1.96) |
| <1x/Week | 1.18 | (0.83,1.67) | 1.15 | (0.81,1.63) |  | 1.21 | (0.84,1.73) | 1.14 | (0.80,1.63) |
| No Use | 1.00 |  | 1.00 |  |  | 1.00 |  | 1.00 |  |
| Any Use in Pregnancy | 1.40 | (1.04,1.90) | 1.33 | (0.98,1.80) |  | 1.39 | (0.99,1.95) | 1.26 | (0.90,1.77) |
| Wald Tests of  Maternal-Paternal |  |  |  |  |  |  |  |  |  |
| >1x/Week | 0.90 |  | 0.59 |  |  | 0.80 |  | 0.82 |  |
| 1x/Week Weekly | 0.28 |  | 0.89 |  |  | 0.80 |  | 0.55 |  |
| <1x/Week | 0.31 |  | 0.52 |  |  | 0.97 |  | 0.92 |  |
| Any Use in Pregnancy | 0.41 |  | 0.44 |  |  | 0.63 |  | 0.67 |  |

**Supplementary Table S7** Coefficient Estimates of the Associations between Maternal and Paternal Cannabis Use and Cognitive Outcomes, with and without adjustment for covariates, two-parent models, ALSPAC imputed sample

|  | IQ | | | |  | GCSE Score | | | |  | SDQ Hyperactivity | | | |
| --- | --- | --- | --- | --- | --- | --- | --- | --- | --- | --- | --- | --- | --- | --- |
|  | Unadjusted | | Adjusted | |  | Unadjusted | | Adjusted | |  | Unadjusted | | Adjusted | |
| Maternal Cannabis | b | 95% CI | b | 95% CI |  | b | 95% CI | b | 95% CI |  | b | 95% CI | b | 95% CI |
| >1x/Week | -1.82 | (-6.63,2.99) | 0.43 | (-3.97,4.82) |  | -26.4 | (-46.6,-6.2) | -8.4 | (-25.6,8.9) |  | 0.40 | (-0.29,1.08) | -0.04 | (-0.72,0.64) |
| 1x/Week Weekly | 0.37 | (-3.32,4.07) | 0.92 | (-2.55,4.39) |  | -10.2 | (-27.1,6.6) | -2.0 | (-16.2,12.3) |  | 0.30 | (-0.22,0.82) | 0.08 | (-0.44,0.60) |
| <1x/Week | 0.53 | (-2.61,3.67) | 0.76 | (-2.13,3.65) |  | -8.5 | (-22.1,5.1) | -0.9 | (-12.3,10.6) |  | -0.14 | (-0.53,0.25) | -0.29 | (-0.66,0.08) |
| No use | 0.00 |  | 0.00 |  |  | 0.0 |  | 0.0 |  |  | 0.00 |  | 0.00 |  |
| Any Use in Pregnancy | -1.47 | (-4.37,1.43) | 0.18 | (-2.44,2.81) |  | -19.2 | (-32.0,-6.3) | -4.4 | (-15.6,6.8) |  | 0.26 | (-0.14,0.65) | -0.07 | (-0.46,0.32) |
| Paternal Cannabis | |  |  |  |  |  |  |  |  |  |  |  |  |  |
| >1x/Week | -0.34 | (-3.32,2.63) | 0.94 | (-1.70,3.57) |  | -10.3 | (-25.8,5.7) | 0.2 | (-11.8,12.2) |  | 0.13 | (-0.26,0.52) | -0.04 | (-0.38,0.30) |
| 1x/Week Weekly | -1.50 | (-4.82,1.81) | -0.23 | (-3.10,2.64) |  | -18.9 | (-34.5,-3.4) | -10.1 | (-22.5,2.3) |  | 0.32 | (-0.13,0.77) | 0.19 | (-0.24,0.61) |
| <1x/Week | 0.32 | (-1.80,2.43) | 0.50 | (-1.21,2.22) |  | -1.7 | (-12.2,8.8) | -0.1 | (-8.3,8.1) |  | 0.13 | (-0.14,0.39) | 0.12 | (-0.13,0.37) |
| No Use | 0.00 |  | 0.00 |  |  | 0.0 |  | 0.0 |  |  | 0.00 |  | 0.00 |  |
| Any Use in Pregnancy | 0.49 | (-1.64,2.62) | 1.33 | (-0.46,3.12) |  | -7.4 | (-18.3,3.6) | 0.2 | (-8.3,8.7) |  | 0.13 | (-0.13,0.39) | 0.01 | (-0.23,0.24) |
| Wald Tests of |  |  |  |  |  |  |  |  |  |  |  |  |  |  |
| Maternal-Paternal |  |  |  |  |  |  |  |  |  |  |  |  |  |  |
| >1x/Week | 0.92 |  | 0.89 |  |  | 0.3 |  | 0.5 |  |  | 0.56 |  | 1.00 |  |
| 1x/Week Weekly | 0.52 |  | 0.66 |  |  | 0.5 |  | 0.4 |  |  | 0.96 |  | 0.77 |  |
| <1x/Week | 0.67 |  | 0.86 |  |  | 0.5 |  | 0.9 |  |  | 0.31 |  | 0.08 |  |
| Any Use in Pregnancy | 0.37 |  | 0.54 |  |  | 0.3 |  | 0.6 |  |  | 0.64 |  | 0.77 |  |
